# The Existence of Airborne Mercury Nanoparticles

**DOI:** 10.1038/s41598-019-47086-8

**Published:** 2019-07-24

**Authors:** Avik. J. Ghoshdastidar, Parisa A. Ariya

**Affiliations:** 1 Department of Chemistry, McGill University, 801 Sherbrooke St. W., Montreal, QC H3A 2K6 Canada; 2 Department of Atmospheric and Oceanic Sciences, McGill University, 805 Sherbrooke St. W., Montreal, QC H3A 0B9 Canada

**Keywords:** Atmospheric chemistry, Mass spectrometry, Element cycles

## Abstract

Mercury is an important global toxic contaminant of concern that causes cognitive and neuromuscular damage in humans. It is ubiquitous in the environment and can travel in the air, in water, or adsorb to soils, snow, ice and sediment. Two significant factors that influence the fate of atmospheric mercury, its introduction to aquatic and terrestrial environments, and its bioaccumulation and biomagnification in biotic systems are the chemical species or forms that mercury exists as (elemental, oxidized or organic) and its physical phase (solid, liquid/aqueous, or gaseous). In this work, we show that previously unknown mercury-containing nanoparticles exist in the air using high-resolution scanning transmission electron microscopy imaging (HR-STEM). Deploying an urban-air field campaign near a mercury point source, we provide further evidence for mercury nanoparticles and determine the extent to which these particles contain two long suspected forms of oxidized mercury (mercuric bromide and mercuric chloride) using mercury mass spectrometry (Hg-MS). Using optical particle sizers, we also conclude that the conventional method of measuring gaseous oxidized mercury worldwide can trap up to 95% of nanoparticulate mercuric halides leading to erroneous measurements. Finally, we estimate airborne mercury aerosols may contribute to half of the oxidized mercury measured in wintertime Montréal urban air using Hg-MS. These emerging mercury-containing nanoparticle contaminants will influence mercury deposition, speciation and other atmospheric and aquatic biogeochemical mercury processes including the bioavailability of oxidized mercury to biota and its transformation to neurotoxic organic mercury.

## Introduction

Mercury has no known beneficial function in the body and can cross both the blood-brain and placental barriers, with known adverse neuromuscular and developmental health effects^1–3^. It is introduced into the environment through natural and anthropogenic processes, with gaseous elemental mercury spending upwards of a year in the atmosphere travelling far from its source of origin^4,5^.

Through chemical conversion to the more water-soluble, bioavailable gaseous oxidized mercury and particulate-bound mercury, it is deposited to terrestrial and aquatic environments. Aquatic microorganisms convert gaseous oxidized mercury to the neurotoxic, organic methylmercury species whose concentrations can bioaccumulate in muscle tissue and increase up to a million-fold in higher order fish^5^.

Anthropogenic point sources can also emit oxidized mercury or mercury present as an inorganic compound. These emissions feature higher proportions of oxidized mercury than what is generally found in the atmosphere, as gaseous elemental mercury typically comprises 90% of total atmospheric mercury or greater^6^. Concerns over the adverse effects of mercury, and the anthropogenic contributions to its release, have prompted over 125 countries to sign the United Nations Environmental Programme’s 2013 Minamata Convention on Mercury.

## The Measurement of Atmospheric Mercury Species

Until recently, determining the chemical forms that oxidized mercury is present as was not feasible analytically, as individual mercury species are only present at ultra-trace concentrations. Atmospheric mercury measurements were restricted to operationally-defined bulk groups of mercury species, such as gaseous elemental mercury (Hg^0^), gaseous oxidized (Hg^2+^ or GOM) mercury, and particulate-bound mercury (Hg^P^).

Gaseous Hg^0^ is measured through double-amalgamation pre-concentration on gold traps, thermal desorption, and detection using cold vapour atomic absorption spectroscopy (CVAFS). With absolute detection limits in the picograms, gaseous Hg^0^ has been measured accurately, and in short time-resolution even in the most remote locations for decades.

The chemical speciation of other atmospheric bulk mercury species, besides gaseous Hg^0^, including all oxidized mercury compounds, has been more challenging. GOM is conventionally collected using KCl-coated annular denuders^7^, where oxidized mercury species (such as mercuric chloride [HgCl_2_]) are complexed as anions ([HgCl_3_]^−^ and [HgCl_4_]^2−^) and incorporated within the KCl matrix^8^.

The denuder is heated above 500 °C resulting in the thermal decomposition of these stable complexes to gaseous Hg^0^ which is subsequently measured by double-amalgamation gold-trap preconcentration CVAFS. As a result of this conversion to gaseous Hg^0^, all information on the chemical form or speciation of the trapped oxidized mercury species is lost^7^.

With advancements in chemical speciation techniques, the chemical species that comprise gaseous oxidized mercury are now being identified. These species include mercuric chloride (HgCl_2_)^9–11^, mercuric bromide (HgBr_2_)^9–11^, organomercury^11–13^, mercuric sulfate (HgSO_4_)^14^, mercuric oxide (HgO)^11,14^, and nitrogen-containing mercury compounds^11,14^. Each chemical compound that comprises gaseous oxidized mercury would have varying degrees of KCl denuder capture, retention and decomposition efficiencies making accurate quantitation of each species and the bulk GOM species difficult. Denuders are also hampered by humidity and ozone interferences that cause mercury loss^11,15^, and the KCl coating itself may also serve as a site of heterogeneous reactions.

Although the existence of particulate-bound mercury (Hg^P^) has been known for decades, mercury-containing nanoparticles has not been seriously considered until recently. Particulate mercury is collected on membrane, fibrous, or quartz filters, with pore sizes ranging between 200 nm and 10 μm, as determined by the investigator^16^.

The filters are either acid-digested or pyrolyzed, and trapped mercury species are reduced to gaseous Hg^0^ which is measured by CVAFS. The chemical species that mercury is present as in these particles and the size distributions of the mercury-containing particles is indeterminable as only the total measure of particulate mercury is measured. Of particular concern are fine particulates, the smallest sizes of which are nanoparticles (<100 nm diameter in one dimension), which are air pollution health hazards and identified by the World Health Organization as a significant cause of premature mortality in humans^17^.

## Airborne Mercury Nanoparticles and its Transformation Pathways

The transformations between gaseous and particulate mercury have a significant impact on the fate of mercury in the atmosphere, its residence time and its eventual deposition to terrestrial and aquatic environments. Particulates provide a surface for elemental gaseous mercury, gaseous oxidized mercury, and other gaseous atoms and molecules to adsorb, collide and react^18,19^. Nanoparticles undergo coagulation processes, growing in size^20,21^, and mercury-containing nanoparticles can act as ice and cloud condensation nuclei promoting dry and wet deposition to marine and terrestrial environments^22^.

GOM has already been shown to adsorb onto KCl and NaCl aerosols, thereby affecting mercury deposition rates^23^. Conversely, the release of gaseous mercuric chloride from inorganic and adipic acid aerosols to the gaseous phase has also been observed^24^, as well as GOM release from fine fraction (<2.5 μm) particulate mercury^7^. The result is a complex interaction of mercury exchange between the gaseous and particulate phases.

**Figure 1:**
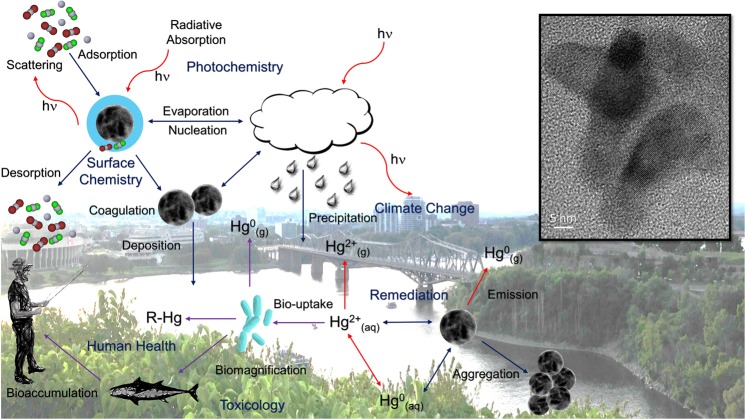
The detection of mercury-containing nanoparticles in urban air and the implications of mercury-containing nanoparticles on the biogeochemistry of atmospheric mercury.

Nano-sized mercury-containing particles, which have higher surface-to-volume ratios, would exhibit more significant release of bioavailable oxidized mercury in atmospheric and aquatic environments depending on the species adsorbed/absorbed to the surface of the particle. Though present in ultra-trace concentrations in air, nano-sized materials are respirable, deposited deep in the lungs, penetrable through tissue^25^, and transportable through the bloodstream where they can be taken up by the placenta^26^. These physical and chemical transformation processes have potentially far-reaching implications for remediation, human health, climate, and biogeochemical modelling (Fig. 1.)

## The Measurement of Nanosized Mercury Particles

No single technique can completely characterize both the physical and chemical properties of nano-sized particles. By using a variety of complementary techniques, many novel to the mercury sciences, the limitations of each are countered by the suite. First, we examine the propensity of the denuder technique for capturing nanoparticulate mercury to determine the potential for contaminated gaseous oxidized mercury measurements, using laboratory synthesized mercury nanoparticles produced at GOM concentrations relevant to stack conditions^27,28^.

Using PFA-based sorbent traps and sampling with and without particle-removing filters upstream, we quantify mercuric bromide and mercuric chloride in the gaseous and particulate phase in wintertime urban air. Tracking atmospheric co-pollutants and meteorological measurements, we examine how these species and factors influence the chemical speciation and phase distribution of oxidized mercury.

Finally, we present the existence of mercury nanoparticles in urban air, using high-resolution scanning transmission electron microscopy with energy-dispersive X-ray spectroscopy (HR-STEM), and its elemental composition. Scanning mobility particle sizers (SMPS), optical particle sizers (OPS), nanoparticle tracking analysis (NTA), high-resolution scanning transmission electron microscopy with energy-dispersive X-ray spectroscopy (HR-STEM) and our recently developed mercury mass spectrometry (Hg-MS)^10^ are employed throughout.

## Results and Discussion

### Characterization of synthetic mercury aerosols to provide a mechanistic understanding

**Figure 2:**
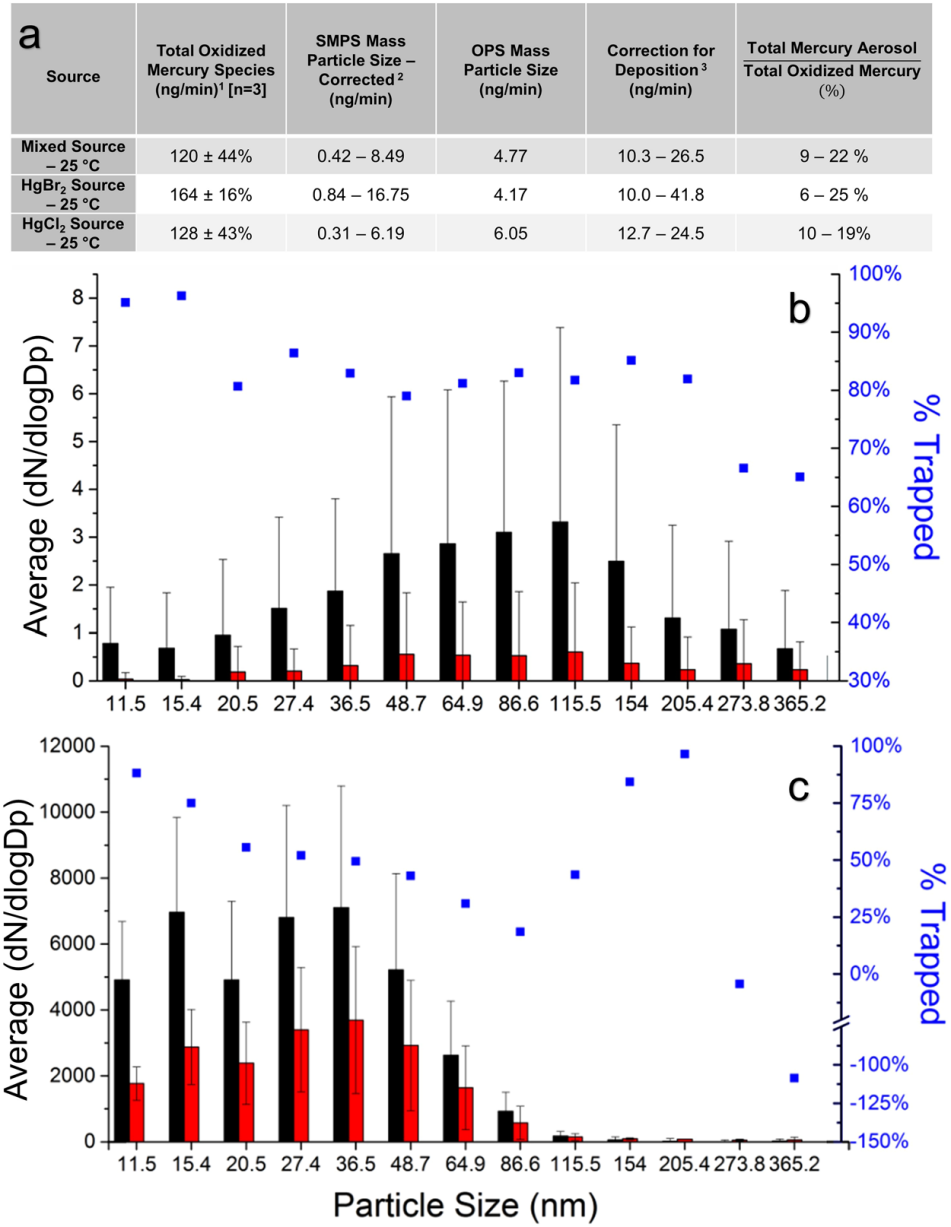
(**a**) Total oxidized mercury from halogenated mercury sources. (1) The KCl Denuder – CVAFS measured the mass of Hg which for HgBr_2_ and HgCl_2_ were corrected to include the mass of the Br and Cl atoms, respectively. The correction for the mixed source was based on the relative ratio between the vapour pressures of mercuric bromide and mercuric chloride at 25 °C. (2) The SMPS unipolar charger typically ionizes between 1% and 20% of particles. As sizing is based on electron mobilities, a charge is required for counting. Ranges for corrected values are given based on this ionization efficiency. (3) At the highest concentrations and highest temperatures, deposition on tubing downstream from the source was observed. A conservative estimate for deposition (1:1) was chosen based on comparable particle counts for deposits and aerosols in Nanoparticle Tracking analysis. (**b**) Denuder capture of synthetic nano-sized mercuric bromide particles produced by vapour condensation method. Particle size distributions for nano-sized mercuric bromide particles produced by vapour flow condensation (shown in black) and those produced by vapour flow condensation and subsequently passed through a KCl denuder (shown in red). Flow streams passed through conductive tubing of equal length before entering the SMPS inlet at a flow rate of 3 LPM. (n = 70). (**c**) Denuder capture of synthetic nano-sized mercuric chloride particles produced from aqueous nebulization. Particle size distributions for nano-sized mercuric chloride particles produced by aqueous solution nebulization (shown in black) and those produced by vapour flow condensation and subsequently passed through a KCl denuder (shown in red). Flow streams passed through conductive tubing of an equivalent length before entering the SMPS inlet at a flow rate of 3 LPM. (n = 70).

Mercury halide aerosols were formed in the laboratory through two methods: vapour flow condensation^29,30^ and by nebulization of aqueous mercuric halides^30,31^. Particulate formation was confirmed using SMPS for sources constructed with a mixture of compositions of mercuric chloride and mercuric bromide (Fig. 2a)^10^.

The majority of particles formed across sources range in size from 50 and 175 nm for vapour flow condensation method, and under 65 nm for nebulization (Fig. 2b). The emission rates (in the hundreds of ng/min) from these vapour flow sources were comparable to others used in laboratory studies to simulate industrial stack gaseous oxidized mercury concentrations^32^, but orders of magnitude higher than would be observed in nature^33^.

The size and morphologies observed from both methods are comparable to oxidized mercury particles formed from vapour phase Hg^0^ reactions with BrO and iodine species^34,35^ and aqueous divalent mercury (Hg^2+^) with thioglycolic acid^36^. The highest number densities were associated with particles 500 nm in size and smaller; particles larger than 1 μm were barely observed.

Micron-sized particulate bound mercury (>3 μm) will not condense from supersaturated oxidized mercury vapour nor as a product of gaseous Hg^0^ photo-oxidation processes and will instead arise from vapour phase Hg absorption on existing fine and coarse aerosols^37^.

When accounting for instrument capabilities and deposition, mercury aerosols formed from vapour flow condensation sources comprise 8–25% of GOM produced from the sources at 20 °C (Fig. 2a), showing that these mercury particles can be emitted from stack conditions, in addition to forming from atmospheric photo-oxidation reactions and other transformation pathways.

As the emissions from stacks will contain many organic and inorganic gaseous and particulate contaminants, any mercury-containing particulates released will be a composite of different forms of mercury on the surface of or incorporated into the bulk of multicomponent particles.

### The capture of mercury aerosols by KCl denuder and contamination of gaseous oxidized mercury measurements

Nanoparticulate mercury, with which we concern ourselves in this study, may be present as elemental or oxidized mercury, absorbed or adsorbed to chemically heterogeneous nanoparticles, or incorporated within the bulk of the particle. These particles may be trapped and measured as particulate bound mercury or deposited through diffusion or electrostatic losses, on the denuder or sampling train surfaces as shown in Fig. 2b,c.

Mercuric bromide and mercuric chloride nanoparticles, formed through vapour flow condensation and nebulization of mercury halide aqueous solutions, were exposed to a manual KCl denuder, the conventional method for capturing gaseous oxidized mercury. While particles larger than 2.5 μm are removed using an impactor, smaller micron and sub-micron particles will travel unrestricted due to laminar flow conditions^15^. However, we found the KCl denuder readily captured nano-sized mercury aerosols at flow rates of 3 litres per minute (LPM) and higher in comparison to flow through a similar length of conductive tubing designed for particulate transmission. In particular, between 70% and 95% of the smallest nanoparticles (>50 nm) were trapped undergoing diffusion related losses^38^ (Fig. 2) with particle transmission efficiency increasing with particle size.

Significant evaporation of particles, as a cause for the decrease particulate concentrations, cannot entirely be ruled out though had evaporation been occurring, we may expect that the particulate size distribution would shift towards smaller particles.

For larger nano-sized particles, the denuder trapping efficiency was sometimes negative, suggesting coagulation of smaller particles to form larger particles or the dislodging of particles from the KCl coating. While the diffusion of nanosized particles is assumed to play a role in denuder capture, the aerosol diffusion coefficient is still orders of magnitude smaller than the gaseous diffusion coefficient of gaseous oxidized mercury species (>0.1 cm^2^/s)^39^.

### Mercury halide aerosols in urban air and the influence of urban air co-pollutants

We performed complementary measurements of mercuric halides in urban air in Montreal, using PFA sorbent traps^10^ with and without upstream filtration. By using filters upstream from the trap, an estimation of the particulate portion of GOM is assessed given the near complete retention of particles by PTFE filters^30^. The entire filter manifold was heated to 50 °C to reduce the adsorption of gaseous oxidized mercury or condensation of water on the filter during sampling^15^. Downstream, PFA sorbent traps have been shown to capture gaseous mercuric halides and nanoparticulate mercuric halides but also serve as a site for the coagulation of larger nano-sized particles.

**Figure 3:**
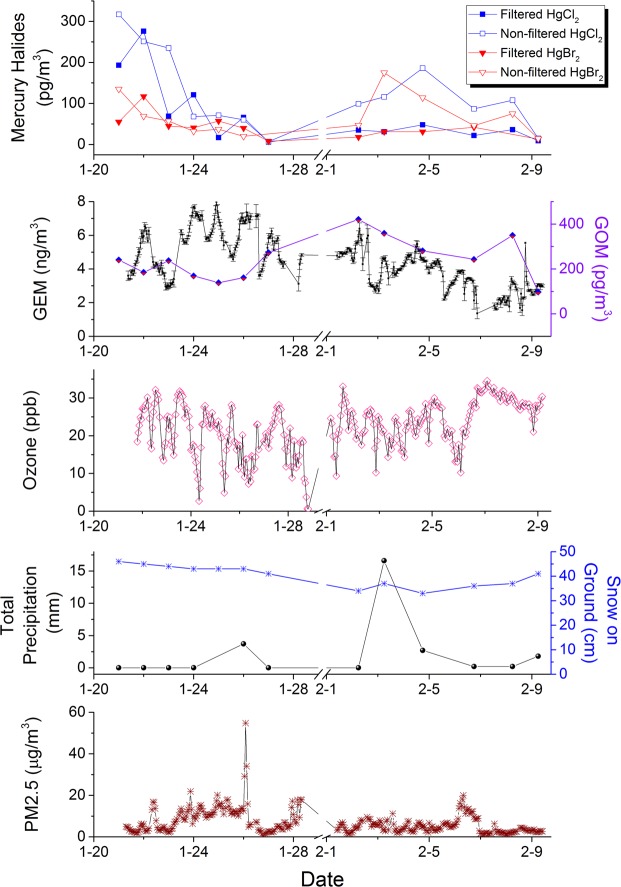
Non-filtered and filtered mercuric halides concentrations by PFA-preconcentration mercury mass spectrometry in Montreal urban air. Gaseous oxidized mercury (GOM) measurements by KCl denuder and gaseous elemental mercury (GEM) measurements by CVAFS. Ozone, total precipitation, snow on the ground and PM_2.5_ measurements from 400 m away. gaseous elemental mercury is strongly negatively correlated with ozone (ρ = −0.67, p = 0.01) and moderately correlated with PM_2.5_ (ρ = −0.57, p = 0.04).

Concentrations of mercuric chloride ranged from undetected to upwards of 300 pg/m^3^, while mercuric bromide peaked at 175 pg/m^3^ (Fig. 3). These concentrations are an order of magnitude higher than are typically found in urban environments using KCl-denuder CVAFS measurements. However, in inter-comparison studies with cationic exchange membrane mercury monitors, the denuder system has been shown to underestimate GOM measurements by at least 1.6 fold^40^.

Despite some higher concentration mass spectrometry measurements of mercuric chloride and mercuric bromide, we found these measurements to be comparable with concurrent manual denuder measurements of gaseous oxidized mercury^33^, and with previous GOM and mercuric halide measurements taken at the site^10^.

At most gaseous oxidized mercury typically consisted of 10% of total atmospheric mercury and the gaseous Hg^0^ values were between two and four times the average found in North American urban centres. As mentioned, the chosen sampling site is a research facility, where mercury is readily used, and the gaseous elemental mercury inlet and denuder were approximately 30 metres from an exhaust stack. The concentrations of mercuric chloride and bromide may be high due to both mercury and halogenated solvents used at the site. The results are comparable to Deeds *et al*. in 2015 where both species (HgCl_2_ and HgBr_2_) were measured at the site^10^.

Chemical speciation of particulate mercury has thus far been approached through leaching of mercury during wet digestion procedures where bulk operational species are defined by their propensity to be extracted under certain digest solution conditions^41–44^. More recently, thermo-desorption is being used, in conjunction with the thermal desorption profiles of inorganic mercury standards, to screen for adsorbed, absorbed and mineral matrix incorporated particulate mercury species^45^. While mass spectrometry has the benefit of providing exact chemical speciation, without adequate separation, there is the potential for artifacts from either ion-source or desorption stage reactions with interferents.

### Quantitation of mercury-bound particles in the air

When differences between non-filtered and filtered measures of mercuric halides were significant, the estimation of maximum mercury aerosols typically ranged between 60% and 80% (Fig. S1).

While the use of filters is a conventional aerosol sampling technique, both positive and negative biases will arise from the heterogeneous reactions of gaseous mercuric species with any aerosols trapped on the filter, the trapping of gaseous species in the filter media, or the volatilization of gaseous mercury from aerosols or filter media^16^. In contrast to fewer oxidative gaseous species and less available sunlight in the winter, colder temperatures will favour greater partitioning to the condensed phase^46^.

Non-filtered gaseous and particulate mercuric bromide (HgBr_2_) showed a moderate correlation^47^ (0.60 < ρ < 0.75) with GOM measurements. (ρ = 0.72, p = 0.008) There was also a strong correlation (0.75 < ρ < 0.85) between filtered and non-filtered HgCl_2_ (ρ = 0.68, p = 0.01) and a moderate correlation between the filtered HgBr_2_ and both non-filtered (ρ = 0.62, p = 0.03) and filtered HgCl_2_ (ρ = 0.60, p = 0.04). Moreover, we observe a decrease in GOM measurements following two precipitation events. As diurnal variations in atmospheric pollutants will influence aerosol concentrations^46^, these measures have been included for comparison to the mercuric halide measurements including NO_x_, SO_2_ and humidity (Fig. S2).

### Recognizing the contribution of mercury nanoparticles to current gaseous oxidized mercury measures

With improvements in cut-points for cascade impactors, nanoparticulate mercury, with diameters as small as 30 nm, have been measured^48^. Atmospheric relevant nanoparticulate bound mercury is expected to be heterogeneous in phase and chemical composition. The smallest nanoparticles may contaminate denuder measurements of GOM through diffusion losses and evaporation of volatile mercury species from these nanoparticles.

The contribution of nanoparticles to denuder GOM measurements is an issue that is important for the accuracy of atmospheric oxidized mercury measurements. At the moment, this critical category of mercury-containing nanoparticles, is not considered in modelling or mercury field measurements worldwide.

**Figure 4:**
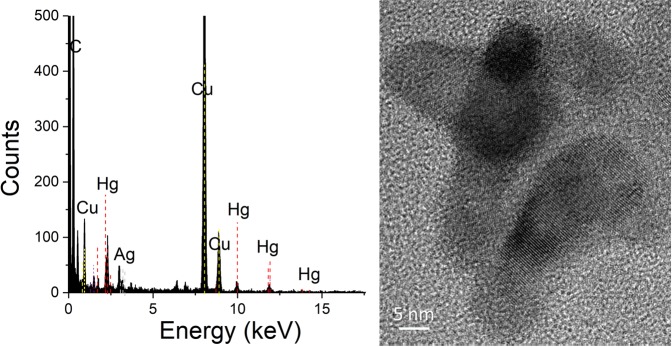
High resolution scanning transmission electron microscopy image of a 10 nm diameter mercury-containing nanoparticle collected in Montreal urban air with EDS indicating the presence of mercury and silver. The prominent Copper peak cannot be distinguished from an artefact resulting from the Formvar-carbon coated copper grid.

We demonstrate the existence of nanosized mercury in the air (Fig. 4). Aerosols were captured using a micro-orifice uniform deposit impactor (MOUDI) with four sub-micron stages. Interestingly, the sub-micron particulate mercury concentrations were on the order of 50–100 pg/m^3^ with the highest concentrations at the lowest cut-off of 180 nm, which is unusual as the accumulation mode for particulate bound mercury occurs around 450 nm.

These concentrations are a magnitude higher than average total mercury particulate levels observed in urban centers^19^. Analyzing TEM grids adhered to the MOUDI stages, we find mercury and silver by EDS in an HR-STEM image of a 10 nm mercury nanoparticle sampled in ambient air at the site (Fig. 4). With the development of nano-MOUDI instruments, further study for smaller nanoparticulate mercury cut-offs is warranted.

### Conclusions and Implications

In conclusion, we have shown the presence of nanoparticulate mercury compounds in urban air and demonstrated that current methods of measuring gaseous oxidized mercury are unable to separate gaseous mercury species and mercury-containing nanoparticles.

The existence of heterogeneous mercury-containing nanoparticles is supported by differences we observe in filtered vs non-filtered mercury mass spectrometry data, nano-sized particulate mercury collection by MOUDI, and TEM images of mercury nanoparticles in ambient air. As a result, the operational field and modelling definitions of particulate and reactive gaseous mercury will need to reflect the emerging contribution of mercury nano-sized particles.

Nanoparticulate mercury will affect physical processes such as wet and dry deposition, atmospheric transport, and chemical processes including plume chemistry, heterogeneous oxidation and reduction processes, surface uptake and catalytic reactions. Furthermore, the incorporation of mercury-bound nanoparticles in the biogeochemical cycling of mercury, and exploring the impact of nanoparticulate mercury in aqueous environments on bio-uptake, bioaccumulation and biomagnification is recommended. Such studies will be crucial to accurate modelling of the fate, assessment and regulation of the ecological and human health risks associated with atmospheric mercury.

## Methods

### Synthetic mercury aerosol sources

Anhydrous mercuric bromide and mercuric chloride, 10 mesh beads, of 99.999% purity were obtained from Sigma Aldrich Canada Co. (Oakville, ON) and used to construct mercury halide sources in ¼ inch I.D. x ^3^/_64_ inch O.D. Swagelok PFA Teflon (Solon, OH) tubing held in place by PTFE frits. Concentrations from the sources for total oxidized mercury and total particulates are provided in Fig. 2a.

High purity nitrogen, used as a carrier gas, was produced using a Peak Scientific NM32LA Nitrogen Generation (Billerica, MA) and was scrubbed of hydrocarbons using Big Hydrocarbon Trap from Agilent Technologies (Mississauga, ON) and particles using high chemical resistance – low flow PVDF filters from Parker Balston (Haverhill, MA). Humidity in the carrier gas line was verified to below 1% using an Aginova iCelsius wireless hygrometer (Mason, OH) and particle-free carrier gas was confirmed before each experiment using a TSI CPC 3007 condensation counter (Shoreview, MN). The carrier gas was heated using an Omega low-flow inline heater, and flow rates maintained using Omega acrylic rotometers (Laval QC).

### Particle number density and distribution of synthetic mercury aerosols

Complementary techniques were used to determine the aerodynamic particle number density and size distribution of synthetic mercury aerosols from 10 nm to 10 μm. The size distributions of synthetic aerosols from 10 nm to 300 nm (in 13 bins) were obtained, over 1 minute scans, using a differential mobility-based Nanoscan SMPS Nanoparticle Sizer 3910 from TSI Incorporated (Shoreview, MN), and of aerosols from 300 nm to 10 μm (in 16 bins), using an Optical Particle Sizer (OPS) 3330.

Conductive tubing was used to prevent electrostatic particle losses in the connections between sources and sizing instruments. The inlet conditioner on the SMPS removes large particles from the aerosol stream and multiple charges applied to the particle can result in lower accuracy at larger sizes. Flow rates through the sources were maintained at 1 L/min, and temperature monitored using an Extech Instruments HD200 Differential Thermometer Datalogger (Nashua, NH).

### Atmospheric pressure chemical ionization-mercury mass spectrometry (Hg-MS)

Mercuric bromide and mercuric chloride were measured using an Agilent 6140 single quadrupole mass spectrometer with an atmospheric pressure chemical ionization source^10^. Samples were collected on in-house made PFA-Teflon traps, heated at 200 °C for 1.2 minutes and desorbed into the APCI source inlet, which had been modified for aerosol and gaseous analysis, using a 1% sulphur hexafluoride in isobutane mixture carrier gas.

In the ion source, sulphur hexafluoride decomposes to form SF_5_^+^ and F^−^ resulting in charge transfer to the target mercuric halides through negatively charged fluoride adduct formation ([M + 19]^−^ corresponding to m/z = 291 for HgCl_2_ + F and m/z = 381 for HgBr_2_ + F). Mercuric bromide, mercuric chloride and mercuric bromochloride were detected as fluoride complexes at m/z 287–295, 331–341, and 388–396, respectively. The mercuric bromochloride species is likely an artifact of ion source reactions^10^. The corona current, capillary voltage and vaporizer (APCI inlet) temperature were set to 30 μA, 750 V, and 200 °C, respectively. N_2_ drying gas flow rate was maintained at 5 L/min and heated to 200 °C. The fragmentor voltage was set to 90 V. These source parameters were optimized across a range of steps and are described by Deeds *et al*.^10^.

### Cold vapour atomic fluorescence spectroscopy

Gaseous elemental mercury was measured using a modified-for-gas sampling Tekran Series 2600 Analysis System (Toronto, ON) with dual stage gold preconcentration with samples acquired every 5 minutes except two hours daily as gaseous oxidized mercury was being measured using a manual denuder and subsequently blanked. Gaseous oxidized was measured manually using KCl-coated annular quartz denuder. The denuder was held at 50 °C during sampling and heated to 525 °C to thermally decompose trapped species to gaseous elemental mercury^7^. The system was calibrated daily using a saturated source of mercury under N_2_.

### Urban air campaign

For urban air samples, samples were gathered daily between January 21^st^, 2016 and February 9^th^, 2016 with two alternating sets of six PFA-traps drawing 1 LPM for roughly 24 hour intervals except for two 48 hour trials between February 1^st^ and 3^rd^ and February 3^rd^ and 5^th^. Traps were calibrated over a four-point calibration with detection limits and sensitivities shown in Table S2 (n = 12). Mercuric bromide and mercuric chloride were measured as fluoride complexes formed from the decomposition of SF_6_ carrier used to desorb these compounds into the APCI ion source.

Given the potential for interferents to mask detection, chemical detection of mercuric bromide or mercuric chloride required either 3 qualifier-to-quantifier ratios within 20% of the theoretical value or 2 qualifier-to-quantifier ratios within 10% of the theoretical value. Concurrent gaseous oxidized mercury samples were drawn for the same sampling periods, in addition to gaseous elemental mercury measurements using the CVAFS.

Additional chemical contaminants (NO_x_ = NO + NO_2_, SO_2_, ozone, PM_2.5_) and meteorological measurements (temperature, relative humidity, snow on ground) were collected from the City of Montreal’s Station 61 – Maisonneuve located 500 m from the mercuric halide sampling site. To remove particulates, 0.45 um PTFE filters from Corning Incorporated (Corning, NY) were attached to the inlet of the PFA-traps and heated to reduce absorption of gaseous oxidized mercury. The experimental set-up is depicted in Fig. S4.

Nano-sized particulate mercury was collected using an M-100 micro-orifice uniform deposit impactor (MOUDI) from MSP Corporation (Shoreview, MN) with 4-submicron stages with aerodynamic diameter cut-points of 1.0 μm, 0.55 μm, 0.21 μm and 0.18 μm. The MOUDI was situated atop a university research building within 30 meters of building exhaust output. Samples were collected on 0.45 μm quartz filters for 24 hours with an inlet flow rate of 30 L/min and digested overnight with 0.5% (v/v) monobromochloride (BrCl) before reduction by 60 μL of 20% (w/v) stannous chloride (SnCl_2_). The resulting gaseous elemental mercury was purged, pre-concentration using dual-stage gold trap amalgamation and analysis by a modified Tekran Series 2600 Analysis System (Toronto, ON).

### High-resolution scanning transmission electron microscopy analysis of ambient air nanoparticles

200 mesh Formvar-carbon coated copper TEM grids and 400 mesh Formvar-coated copper TEM grids were obtained from Electron Microscope Sciences (Hatfield, PA) and SPI Supplies (West Chester, PA), and placed on the stages of the MOUDI impactor for 24 hours to collect particulate matter for analysis with high-resolution scanning transmission electron microscopy. Samples were analyzed using FEI Tecnai G^2^ F20 kV Cryo-STEM with EDAX Octane T Ultra W/Apollo XLT_2_ SDD and TEAM EDS Analysis System (Hillsboro, OR).

## Supplementary information

Supplementary Info

## References

[1] Tchounwou PB, Ayensu WK, Ninashvili N, Sutton D (2003). Review: Environmental exposure to mercury and its toxicopathologic implications for public health. Environ Toxicol.

[2] Aschner M, Aschner JL (1990). Mercury neurotoxicity: mechanisms of blood-brain barrier transport. Neuroscience & Biobehavioral Reviews.

[3] Atwell L, Hobson KA, Welch HE (1998). Biomagnification and bioaccumulation of mercury in an arctic marine food web: insights from stable nitrogen isotope analysis. Canadian Journal of Fisheries and Aquatic Sciences.

[4] Pacyna EG, Pacyna JM, Steenhuisen F, Wilson S (2006). Global anthropogenic mercury emission inventory for 2000. Atmospheric environment.

[5] Schroeder WH, Munthe J (1998). Atmospheric mercury—an overview. Atmos Environ.

[6] Subir M, Ariya PA, Dastoor AP (2012). A review of the sources of uncertainties in atmospheric mercury modeling II. Mercury surface and heterogeneous chemistry – A missing link. Atmos Environ.

[7] Landis MS, Stevens RK, Schaedlich F, Prestbo EM (2002). Development and Characterization of an Annular Denuder Methodology for the Measurement of Divalent Inorganic Reactive Gaseous Mercury in Ambient Air. Environ Sci Technol.

[8] Sommar J, Feng X, Gardfeldt K, Lindqvist O (1999). Measurements of fractionated gaseous mercury concentrations over northwestern and central Europe, 1995-99. J Environ Monitor.

[9] Jones CP, Lyman SN, Jaffe DA, Allen T, O’Neil TL (2016). Detection and quantification of gas-phase oxidized mercury compounds by GC/MS. Atmos Meas Tech.

[10] Deeds DA (2015). Development of a Particle-Trap Preconcentration-Soft Ionization Mass Spectrometric Technique for the Quantification of Mercury Halides in Air. Anal Chem.

[11] Huang JY, Miller MB, Edgerton E, Gustin MS (2017). Deciphering potential chemical compounds of gaseous oxidized mercury in Florida, USA. Atmos Chem Phys.

[12] Schroeder WH, Jackson RA (1987). Environmental measurements with an atmospheric mercury monitor having speciation capabilities. Chemosphere.

[13] Brosset C, Lord E (1995). Methylmercury in ambient air. Method of determination and some measurement results. Water, Air, Soil Pollut..

[14] Gustin MS (2016). Evidence for Different Reactive Hg Sources and Chemical Compounds at Adjacent Valley and High Elevation Locations. Environ Sci Technol.

[15] Lyman SN, Jaffe DA, Gustin MS (2010). Release of mercury halides from KCl denuders in the presence of ozone. Atmos Chem Phys.

[16] Lu JY (1998). A Device for Sampling and Determination of Total Particulate Mercury in Ambient Air. Anal Chem.

[17] Organization, W. H. Review of evidence on health aspects of air pollution–REVIHAAP Project. *World Health Organization, Copenhagen, Denmark* (2013).

[18] Subir M, Ariya PA, Dastoor AP (2012). A review of the sources of uncertainties in atmospheric mercury modeling II. Mercury surface and heterogeneous chemistry - A missing link. Atmospheric Environment.

[19] Kim P-R, Han Y-J, Holsen TM, Yi S-M (2012). Atmospheric particulate mercury: Concentrations and size distributions. Atmospheric Environment.

[20] Murphy DM, Hudson PK, Thomson DS, Sheridan PJ, Wilson JC (2006). Observations of Mercury-Containing Aerosols. Environ Sci Technol.

[21] Zhang L, Gong S, Padro J, Barrie L (2001). A size-segregated particle dry deposition scheme for an atmospheric aerosol module. Atmos Environ.

[22] Ganguly M, Dib S, Ariya PA (2018). Purely Inorganic Highly Efficient Ice Nucleating Particle. ACS Omega.

[23] Malcolm E (2010). Experimental investigation of the scavenging of gaseous mercury by sea salt aerosol. J Atmos Chem.

[24] Rutter AP, Schauer JJ (2007). The effect of temperature on the gas–particle partitioning of reactive mercury in atmospheric aerosols. Atmos Environ.

[25] Int Panis L (2010). Exposure to particulate matter in traffic: A comparison of cyclists and car passengers. Atmos Environ.

[26] Wick P (2010). Barrier Capacity of Human Placenta for Nanosized Materials. Environ Health Persp.

[27] Jun Lee S (2004). Mercury emissions from selected stationary combustion sources in Korea. Sci Total Environ.

[28] Kotnik J, Horvat M, Mandic V, Logar M (2000). Influence of the Šoštanj coal-fired thermal power plant on mercury and methyl mercury concentrations in Lake Velenje, Slovenia. Sci Total Environ.

[29] Swihart MT (2003). Vapor-phase synthesis of nanoparticles. Current Opinion in Colloid & Interface Science.

[30] Ghoshdastidar, A. J., Ramamurthy, J., Morrisette, M. & Ariya, P. A. Development of Methodology to Generate, Measure and Characterize the Chemical Composition of Oxidized Mercury Nanoparticles. *Anal Bioanal Chem In Revision* [*ABC-00761-2019]* (2019). 10.1007/s00216-019-02279-y 31853601

[31] Nazarenko Y, Rangel-Alvarado RB, Kos G, Kurien U, Ariya PA (2017). Novel aerosol analysis approach for characterization of nanoparticulate matter in snow. Environmental Science and Pollution Research.

[32] Wängberg I (2003). Atmospheric mercury near a chlor-alkali plant in Sweden. Sci Total Environ.

[33] Sprovieri F, Pirrone N, Ebinghaus R, Kock H, Dommergue A (2010). A review of worldwide atmospheric mercury measurements. Atmos Chem Phys.

[34] Raofie F, Snider G, Ariya PA (2008). Reaction of gaseous mercury with molecular iodine, atomic iodine, and iodine oxide radicals - Kinetics, product studies, and atmospheric implications. Can J Chem.

[35] Raofie F, Ariya PA (2004). Product study of the gas-phase BrO-initiated oxidation of Hg-0: evidence for stable Hg1+ compounds. Environ Sci Technol.

[36] Si L, Ariya PA (2015). Photochemical reactions of divalent mercury with thioglycolic acid: Formation of mercuric sulfide particles. Chemosphere.

[37] Keeler G, Glinsorn G, Pirrone N (1995). Particulate mercury in the atmosphere: its significance, transport, transformation and sources. Water Air Soil Pollut.

[38] Ye Y, Tsai C-J, Pui DYH, Lewis CW (1991). Particle Transmission Characteristics of an Annular Denuder Ambient Sampling System. Aerosol Sci Tech.

[39] Seinfeld, J. H. & Pandis, S. N. *Atmospheric chemistry and physics: from air pollution to climate change*. (John Wiley & Sons, 2012).

[40] Huang JY, Miller MB, Weiss-Penzias P, Gustin MS (2013). Comparison of Gaseous Oxidized Hg Measured by KCl-Coated Denuders, and Nylon and Cation Exchange Membranes. Environ Sci Technol.

[41] Xiu GL (2009). Speciated mercury in size-fractionated particles in Shanghai ambient air. Atmospheric Environment.

[42] Xiu GL (2005). Characterization of size-fractionated particulate mercury in Shanghai ambient air. Atmospheric Environment.

[43] Zverina O, Coufalik P, Komarek J, Gadas P, Sysalova J (2014). Mercury associated with size-fractionated urban particulate matter: three years of sampling in Prague, Czech Republic. Chem Pap.

[44] Cheng N, Duan L, Xiu G, Zhao M, Qian G (2017). Comparison of atmospheric PM2.5-bounded mercury species and their correlation with bromine and iodine at coastal urban and island sites in the eastern China. Atmos Res.

[45] Bełdowska M (2018). Simple screening technique for determination of adsorbed and absorbed mercury in particulate matter in atmospheric and aquatic environment. Talanta.

[46] Lynam MM, Keeler GJ (2005). Artifacts associated with the measurement of particulate mercury in an urban environment: The influence of elevated ozone concentrations. Atmospheric Environment.

[47] Kozak M (2009). What is strong correlation?. Teaching Statistics.

[48] Pyta H, Rogula-Kozlowska W (2016). Determination of mercury in size-segregated ambient particulate matter using CVAAS. Microchem J.

