# Supplementary material for: The Existence of Airborne Mercury Nanoparticles
Source: Sci Rep. 2019 Jul 24;9:10733. doi: 10.1038/s41598-019-47086-8 (PMC6656720; doi:10.1038/s41598-019-47086-8)
Supplement: Supplementary file 1 — Supplementary Info [file 41598_2019_47086_MOESM1_ESM.pdf]

The Existence of Airborne Mercury Nanoparticles

Avik. J. Ghoshdastidar[1] & *Parisa A. Ariya*[1, 2]*

[1]Department of Chemistry and [2]Department of Atmospheric and Oceanic Sciences

McGill University, 801 Sherbrooke St. W., Montreal, QC, Canada, H3A 2K6

*Corresponding author: parisa.ariya@mcgill.ca

Supplementary Information

Tables

Table S1. Summary of GOM and PBM speciation techniques 2
Table S2. Mercury mass spectrometry (Hg-MS) calibration 4

Figures

Figure S1. Mercuric Halides GOM and the contribution of mercury aerosols in Montreal's urban air. 5
Figure S2. Mercuric Halides, NO_x, SO_2, humidity and temperature in Montreal. 6
Figure S3. The vapour pressure of mercuric bromide and mercuric chloride [4,14-18] 7
Figure S4. The experimental setup for the detection of gaseous and particulate mercuric halides with concurrent gaseous oxidized mercury and gaseous elemental mercury measurements 8

Table S1. Summary of GOM and PBM speciation techniques

Expansion of Supplementary Table S1 in *Deeds et al. Anal (2015) Chem, 87, (10) 5109-5116*

Reference	Sample Collection	Sample Preparation	Detection Technique	Form of GOM/PBM Measured
Braman and Johnson, 1974 [1]	HCl treated – Chromosorb-W	Thermal Desorption	DC Discharge Emission Spectroscopy	Gaseous Hg(II) type compounds
	NaOH treated – Chromosorb-W			Gaseous Methylmercury (II) type compounds
	Gold-coated glass beads			Gaseous Dimethylmercury
Stratton and Lindberg, 1995 [2]	HCl Mist Chamber	Stannous Chloride ($SnCl_2$) Reduction	Cold Vapor Atomic Fluorescence Spectroscopy	Reactive Gaseous Mercury [a]
Sommar et al., 1997 [3]	KCl Denuder	Thermal Decomposition	Cold Vapor Atomic Fluorescence Spectroscopy	Divalent Gaseous Mercury
Tong et al., 1999 [4]	None	None	Photo-fragment Fluorescence Spectroscopy	Gaseous $HgBr_2$
Landis et al., 2002 [5]	KCl Denuder	Thermal Decomposition	Cold Vapor Atomic Fluorescence Spectroscopy	Reactive Gaseous Mercury [b]
Olson et al., 2002 [6]	5% MnO_2 Alumina and Activated Carbon Sorbents	Thermal Desorption	Gas Chromatography – Mass Spectrometry	Gaseous $HgCl_2$<br>Gaseous $Hg(NO_3)_2$
Xiu et al. 2005 [7]	Glass Fiber and Quartz Fiber Filter	Successive Digestions using Leaching Solutions	Cold Vapor Atomic Absorption Spectroscopy	Reactive PM<br>Volatile PM<br>Inert PM
Xiu et al. 2009 [8]	Glass Fibre Filter	Successive Digestions using Leaching Solutions	Cold Vapor Atomic Absorption Spectroscopy	Exchangeable PM<br>HCl-soluble PM,<br>Elemental PM<br>Residual PM
Lynam et al., 2010 [9]	Polysulfone Cation-Exchange Membrane	Acid Digestion and Chemical Reduction	Cold Vapor Atomic Fluorescence Spectroscopy	Gaseous Oxidized Mercury
Huang et al. 2013 [10]	Nylon Membrane	Thermal Desorption	Cold Vapor Atomic Fluorescence Spectroscopy	Gaseous Oxidized Mercury

Zverina et al. 2014 [11]	Fibrous Filters	Successive Extractions using Leaching Solutions	Cold Vapor Atomic Absorption Spectroscopy	Water extractable PM,<br>Acid-released PM,<br>Organic bound PM<br>Elemental and complex-bound PM
Deeds et al, 2015 [12]	Particle-based Sorbent Trap	Thermal Desorption	Atmospheric Pressure Chemical Ionization Mass Spectrometry	$HgBr_2$<br>$HgCl_2$
Jones et al., 2016 [13]	Nylon Membrane, PDMS, quartz wool and deactivated fused silica-coated stainless steel	Thermal Desorption with Cryogenic preconcentration focusing	Gas Chromatography – Mass Spectrometry	$HgBr_2$<br>$HgCl_2$

[a] Described as the "fraction of Hg reducible by $SnCl_2$"

[b] Described as "water soluble (divalent) inorganic forms"

Table S2. Mercury mass spectrometry (Hg-MS) calibration

	$HgCl_2$ Detection Limit (pg)	$HgBr_2$ Detection Limit (pg)	Sensitivity – $HgCl_2$ (cnts/pg)	Sensitivity – $HgBr_2$ (cnts/pg)	R^2 – $HgCl_2$	R^2 – $HgBr_2$
Trap 1	16	13	240	480	0.82	0.88
Trap 2	69	61	275	565	0.91	0.90
Trap 3	43	13	280	475	0.95	0.83
Trap 4	39	21	600	805	0.93	0.82
Trap 5	23	16	645	655	0.83	0.91
Trap 6	34	26	390	675	0.90	0.94
Trap 7	16	49	360	765	0.91	0.95
Trap 8	9	7	320	650	0.96	0.89
Trap 9	18	22	515	635	0.88	0.85
Trap 10	13	13	375	810	0.83	0.80
Trap 11	60	56	395	455	0.89	0.91
Trap 12	15	27	470	875	0.92	0.78

Figure S1. Mercuric Halides GOM and the contribution of mercury aerosols in Montreal's urban air.

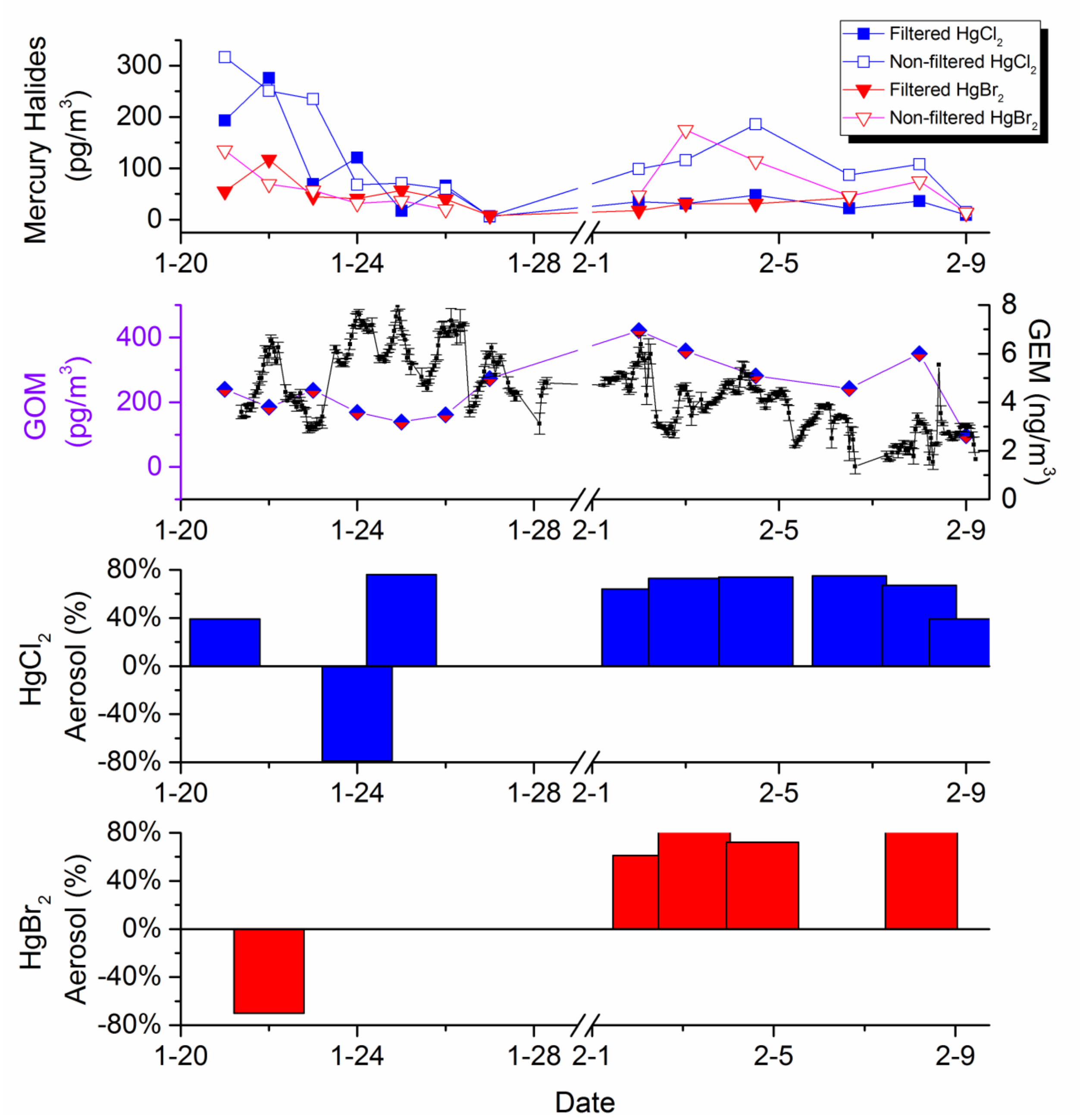

Non-filtered and filtered mercuric halides concentrations by shredded PFA-Teflon preconcentration mercury mass spectrometry in Montreal urban. GOM measurements by KCl denuder and GEM measurements by CVAFS. The percentage of mercuric halide aerosols was estimated by the difference between the filtered and non-filtered mass spectrometry concentration averages where the difference was statistically significant.

Figure S2. **Mercuric Halides, NO_x, SO_2, humidity and temperature in Montreal.**

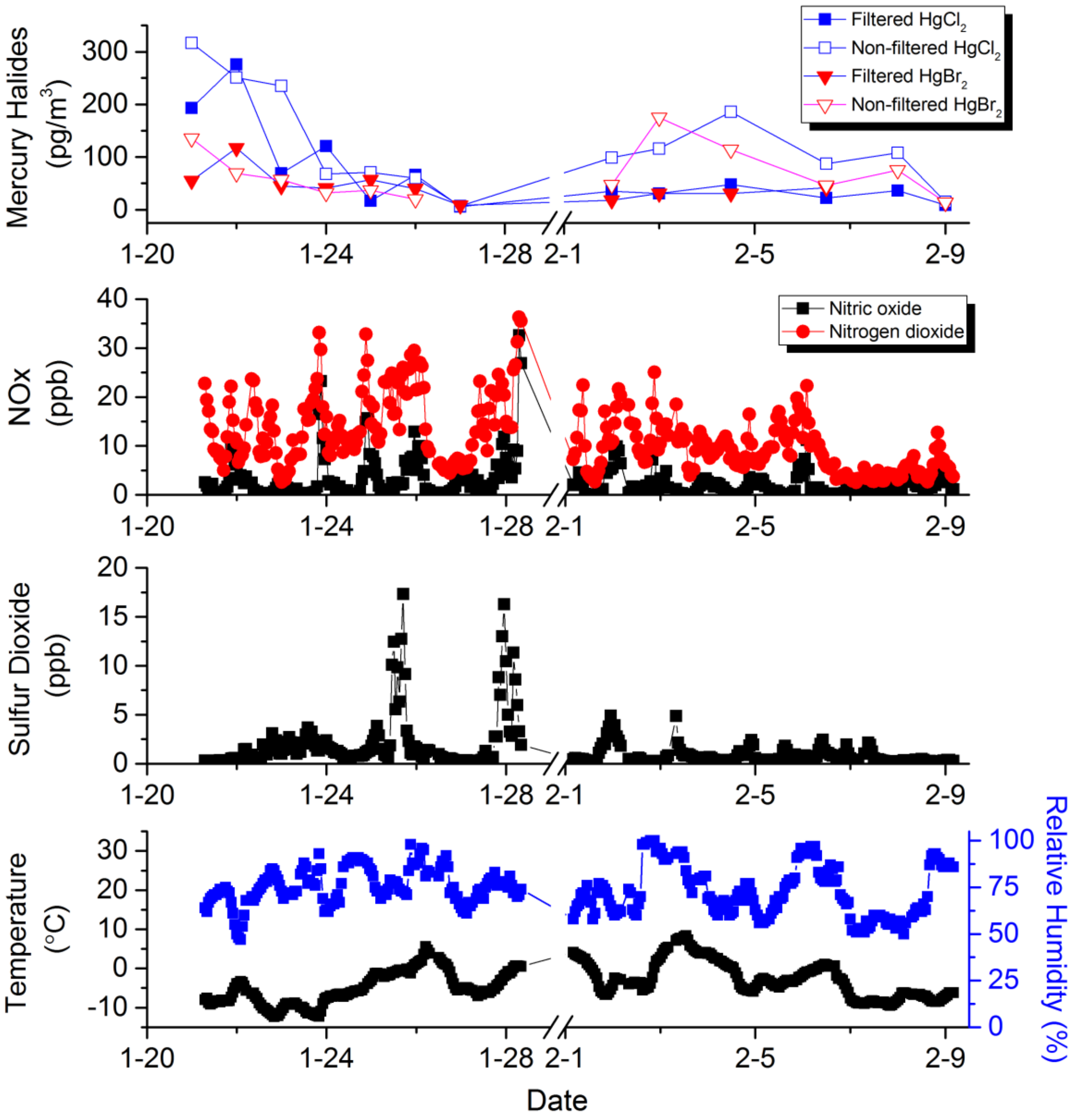

Teflon preconcentration mercury mass spectrometry in Montreal urban air. NOx, sulphur dioxide, relative humidity and temperature measurements were taken 400 m away.

Figure S3. **The vapour pressure of mercuric bromide and mercuric chloride** [4,14-18]

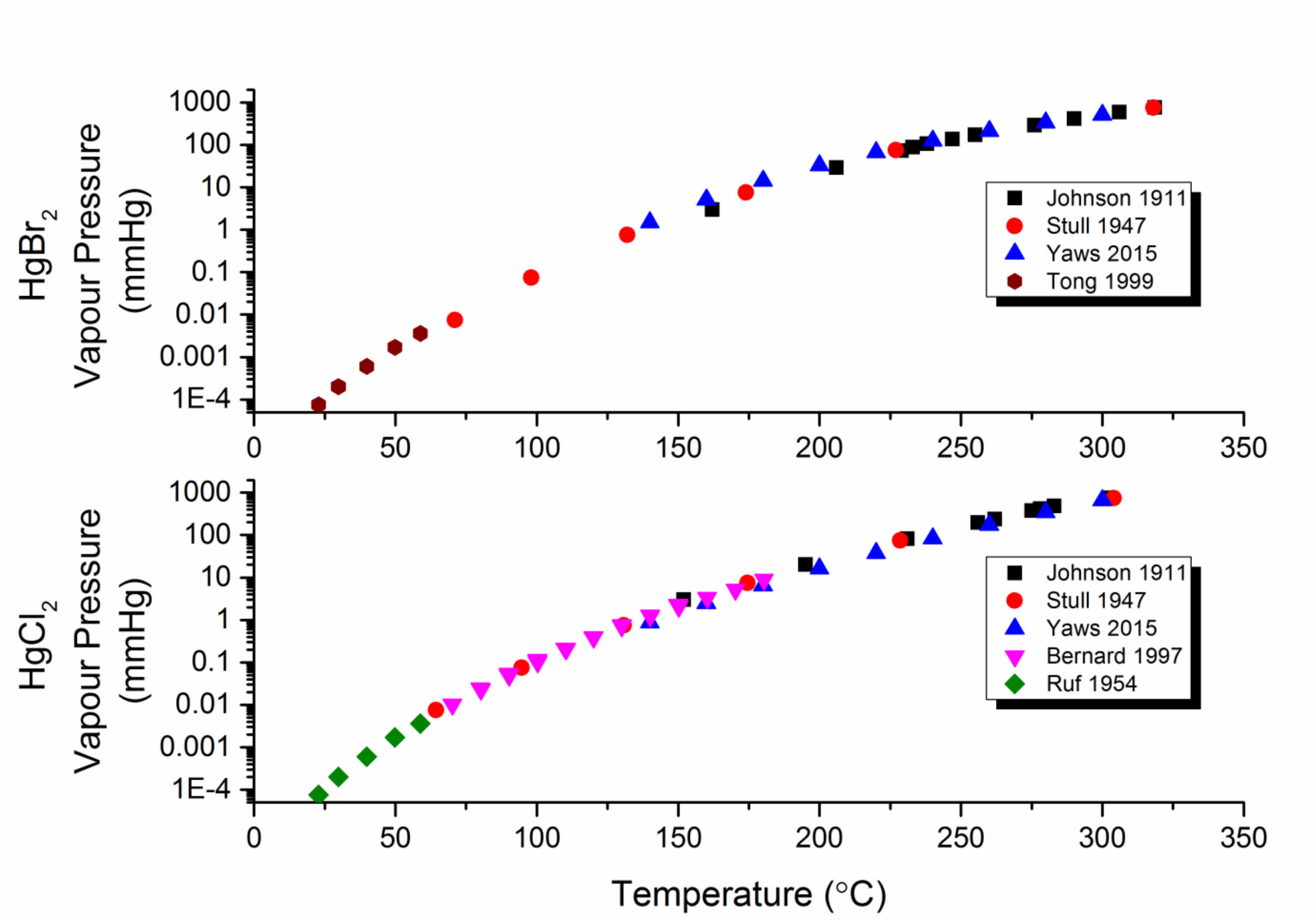

Figure S4. The experimental setup for the detection of gaseous and particulate mercuric halides with concurrent gaseous oxidized mercury and gaseous elemental mercury measurements

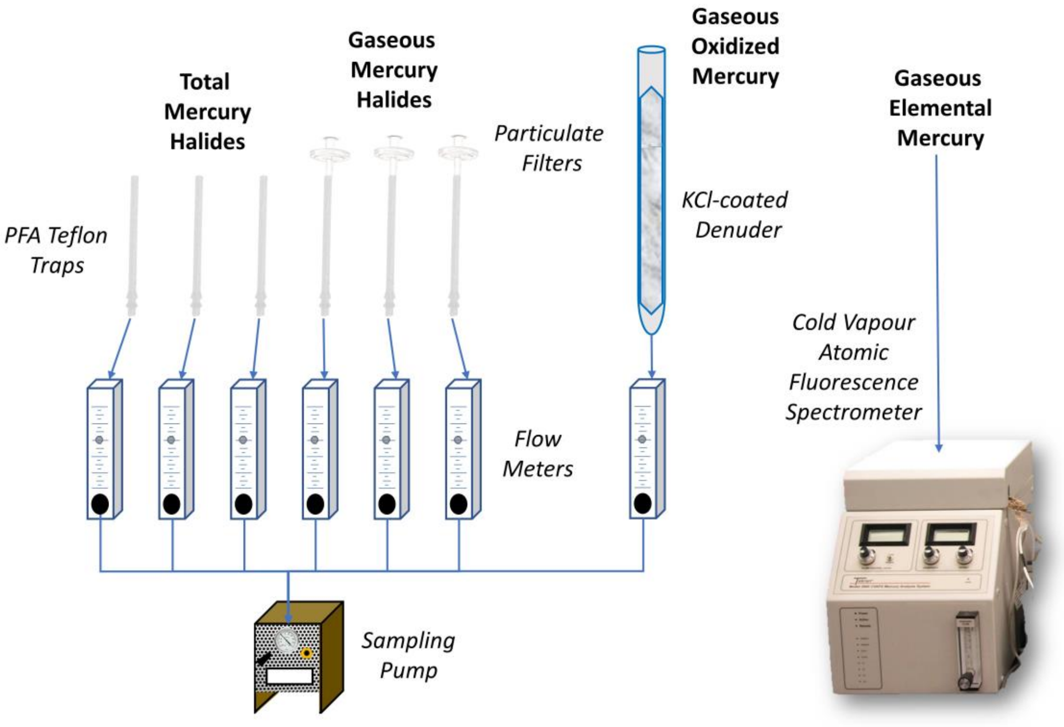

S2. References

1 Braman, R. S. & Johnson, D. L. Selective absorption tubes and emission technique for determination of ambient forms of mercury in air. *Environ Sci Technol* **8**, 996-1003 (1974).

2 Stratton, W. J. & Lindberg, S. E. in *Mercury as a Global Pollutant* (eds DonaldB Porcella, JohnW Huckabee, & Brian Wheatley) Ch. 144, 1269-1278 (Springer Netherlands, 1995).

3 Xiao, Z., Sommar, J., Wei, S. & Lindqvist, O. Sampling and determination of gas phase divalent mercury in the air using a KCl coated denuder. *Fresenius J Anal Chem* **358**, 386-391, doi:10.1007/s002160050434 (1997).

4 Tong, X., Barat, R. B. & Poulos, A. T. Detection of Mercuric Bromide in a Gas Phase Flow Cell by Laser Photofragment Fluorescence Spectroscopy. *Environ Sci Technol* **33**, 3260-3263, doi:10.1021/es9813461 (1999).

5 Landis, M. S., Stevens, R. K., Schaedlich, F. & Prestbo, E. M. Development and Characterization of an Annular Denuder Methodology for the Measurement of Divalent Inorganic Reactive Gaseous Mercury in Ambient Air. *Environ Sci Technol* **36**, 3000-3009, doi:10.1021/es015887t (2002).

6 Olson, E., Sharma, R. & Pavlish, J. On the analysis of mercuric nitrate in flue gas by GC–MS. *Anal Bioanal Chem* **374**, 1045-1049, doi:10.1007/s00216-002-1602-6 (2002).

7 Xiu, G. L. *et al.* Characterization of size-fractionated particulate mercury in Shanghai ambient air. *Atmospheric Environment* **39**, 419-427, doi:https://doi.org/10.1016/j.atmosenv.2004.09.046 (2005).

8 Xiu, G. L. *et al.* Speciated mercury in size-fractionated particles in Shanghai ambient air. *Atmospheric Environment* **43**, 3145-3154, doi:10.1016/j.atmosenv.2008.07.044 (2009).

9 Lyman, S. N., Gustin, M. S. & Prestbo, E. M. A passive sampler for ambient gaseous oxidized mercury concentrations. *Atmos Environ* **44**, 246-252, doi:http://dx.doi.org/10.1016/j.atmosenv.2009.10.008 (2010).

10 Huang, J., Miller, M. B., Weiss-Penzias, P. & Gustin, M. S. Comparison of Gaseous Oxidized Hg Measured by KCl-Coated Denuders, and Nylon and Cation Exchange Membranes. *Environ Sci Technol* **47**, 7307-7316, doi:10.1021/es4012349 (2013).

11 Zverina, O., Coufalik, P., Komarek, J., Gadas, P. & Sysalova, J. Mercury associated with size-fractionated urban particulate matter: three years of sampling in Prague, Czech Republic. *Chem Pap* **68**, 197-202, doi:10.2478/s11696-013-0436-3 (2014).

12 Deeds, D. A. *et al.* Development of a particle-trap preconcentration-soft ionization mass spectrometric technique for the quantification of mercury halides in air. *Anal Chem* **87**, 5109-5116, doi:10.1021/ac504545w (2015).

13 Jones, C. P., Lyman, S. N., Jaffe, D. A., Allen, T. & O'Neil, T. L. Detection and quantification of gas-phase oxidized mercury compounds by GC/MS. *Atmos. Meas. Tech.* **9**, 2195-2205, doi:10.5194/amt-9-2195-2016 (2016).

14 Johnson, F. THE VAPOR PRESSURES OF MERCURIC CHLORIDE, BROMIDE AND IODIDE. *J Am Chem Soc* **33**, 777-781 (1911).

15 Stull, D. R. Vapor Pressure of Pure Substances. Organic and Inorganic Compounds. *Industrial & Engineering Chemistry* **39**, 517-540, doi:10.1021/ie50448a022 (1947).

16 Yaws, C. L. & Satyro, M. A. in *The Yaws Handbook of Vapor Pressure (Second Edition)* 315-322 (Gulf Professional Publishing, 2015).

17 Bernard, L. *et al.* Détermination de la pression de vapeur de HgCl$\mathsf{_2}$ par la méthode d'effusion de Knudsen. *J. Phys. III France* **7**, 311-319 (1997).

18 Ruf, R. & Treadwell, W. D. Zur Kenntnis des Dampfdrucks von Quecksilber(II)-chlorid. *Helvetica Chimica Acta* **37**, 1941-1948, doi:10.1002/hlca.19540370705 (1954).
